# Stenotrophomonas Maltophilia and Urinary Tract Infections: A Systematic Review

**DOI:** 10.7759/cureus.26184

**Published:** 2022-06-21

**Authors:** Zaryab Umar, Salman Ashfaq, Avish Parikh, Usman Ilyas, Allison Foster, Rubal Bhangal, Jawad Khan, Mahmoud Nassar

**Affiliations:** 1 Internal Medicine, Icahn School of Medicine at Mount Sinai, Queens Hospital Center, New York, USA; 2 Internal Medicine, Allama Iqbal Medical College, Lahore, PAK

**Keywords:** trimethoprim-sulfamethoxazole (tmp-smx), nephrologic pathologies, urologic pathologies, nosocomial infection, uti, urinary tract infection, stenotrophomonas maltophilia

## Abstract

Stenotrophomonas maltophilia, a gram-negative bacillus well known to cause respiratory tract infections, is increasingly being reported to cause urinary tract infections (UTI). In our review of the literature comprising six articles, males were more prone to developing UTIs, with the mean age of the patients being 62.5 ±18.9 years. While several risk factors have been associated with the development of the disease, patients with underlying urological or nephrological diseases tend to develop a more severe illness. The organism was sensitive to trimethoprim-sulfamethoxazole (TMP-SMX) in the majority of cases. This systematic review also aims to shed light on the possible mechanisms of resistance adopted by the bacteria, modes of transmission, and strategies to prevent the transmission and development of the disease.

## Introduction and background

Stenotrophomonas maltophilia is a leading cause of nosocomial infections in hospitals worldwide and is often implicated in serious bacterial infections [1,2]. The organism is a gram-negative bacillus, which causes respiratory tract infections worldwide [1,2]. Even though the organism is considered to have low virulence in immunocompetent patients or patients without risk factors, the combination of multiple methods of antimicrobial resistance, the ability to form biofilms on indwelling catheters, and increasing antibiotic resistance significantly increase the possibility of the organism causing a serious infection [2,3]. Stenotrophomonas maltophilia has primarily been described as a respiratory pathogen; however, it has also been found to cause urinary tract infections (UTI), osteomyelitis, meningitis, catheter-associated bacteremia, and endocarditis in patients with certain risk factors [2,4-7]. This article aims to describe the incidence of UTIs induced by this pathogen, the associated risk factors, and the treatment options in such cases.

## Review

Methods

We reviewed the following databases through April 2nd, 2022: Embase, Medline, PubMed, and Web of Science. Keywords included "Stenotrophomonas maltophilia [MeSH Terms]" and "urinary tract infection MeSH" or "UTI". The inclusion criteria were as follows: primary studies, case reports or case series, confirmed cases of Stenotrophomonas maltophilia, and articles written in English. Exclusion criteria included review articles, meta-analyses, systematic reviews, non-English articles, studies on pediatric and pregnant cases, non-peer-reviewed articles, and guidelines. The articles retrieved were screened by a single reviewer who worked independently.

Results

**Figure 1:**
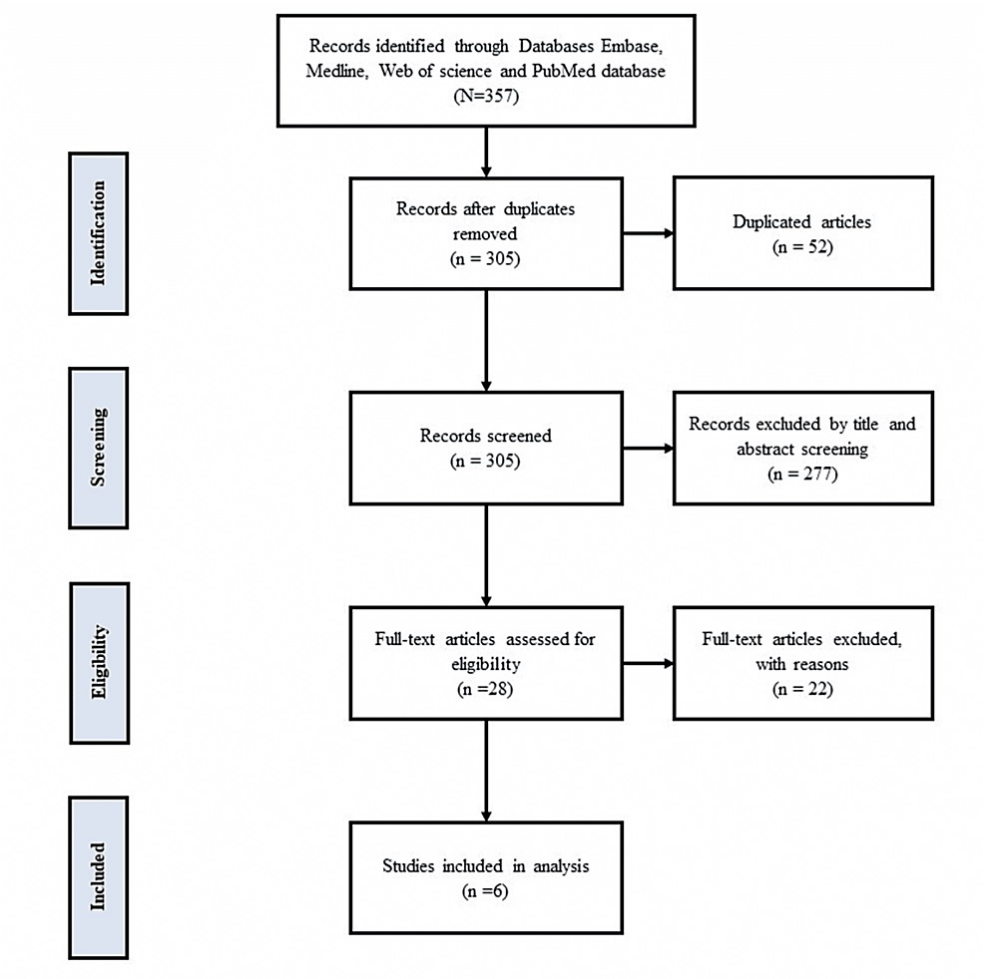
PRISMA flow diagram of the study screening process PRISMA: Preferred Reporting Items for Systematic Reviews and Meta-Analyses

Of the 357 articles identified, 52 were found to be duplicates and were removed. Titles and abstracts of the remaining 305 articles were screened. At this stage, 277 articles were excluded. A full-text screening of the remaining 28 articles resulted in the inclusion of six articles and the exclusion of 22 articles. Figure 1 depicts the Preferred Reporting Items for Systematic Reviews and Meta-Analyses (PRISMA) flow diagram of the study screening process.

There were five (83.3%) male patients and one female patient. The mean age of the patients was 62.5 ±18.9 years (range: 41-89 years). Table 1 summarizes the patient characteristics.

**Table 1:** Summary of patient characteristics PEG: percutaneous endoscopic gastrostomy; UTI: urinary tract infection; COPD: chronic obstructive pulmonary disease; ICU: intensive care unit

Study	Sex	Age (years)	Admission diagnosis/underlying disease	Comorbid disease	Device used	Other risk factors	Nosocomial infection diagnosis	Specimen type	Antibiotic agent
Vaidyanathan et al. (2005) [8]	Male	50	Pyonephrosis caused by a large calculus in the renal pelvis	Tetraplegia		(1) Surgical intervention: nephrostomy, pigtail catheter, double J stent placement, extracorporeal shock wave lithotripsy. (2) Teicoplanin, ciprofloxacin, gentamicin, metronidazole, cefuroxime	Superinfection of perinephric abscess by maltophilia	Collected from nephrostomy tube and pigtail catheter	Teicoplanin, ciprofloxacin, gentamicin, metronidazole, cefuroxime
Savini et al. (2010) [9]	Male	NA	Patient with myelofibrosis undergoing treatment with permanent bladder catheterization	Myelofibrosis	Bladder catheter	(1) Broad-spectrum antibiotics: levofloxacin, amoxicillin/clavulanate, ceftazidime, piperacillin/tazobactam. (2) Hospital stay exceeding 30 days	Asymptomatic growth of maltophilia in the bladder device	Collected from bladder device	Chloramphenicol, rifampin
Petca et al. (2022) [10]	Female	41	Severe left pyonephrosis	COPD, nephroureterolithiasis, and normochromic normocytic anemia; the patient underwent double J stent placement a few months back for high-grade uretero-hydronephrosis		(1) Surgical intervention: exploratory lumbotomy with left nephrectomy, two drainage catheters placed afterward. (2) Meropenem, metronidazole, amikacin		Fluid drained during nephrectomy	Trimethoprim/sulfamethoxazole, aminoglycosides, fluoroquinolones, tetracycline, colistin
Lee et al. (1997) [11]	Male	73	Radical cystoprostatectomy with an ileal conduit for transitional cell carcinoma of the bladder, recurrent UTIs, and nephrolithiasis			(1) ICU stay. (2) Jejunostomy, tracheostomy. (3) Piperacillin, tobramycin, vancomycin, ciprofloxacin, trimethoprim/sulfamethoxazole, and fluconazole. (4) Hospital stay exceeding 30 days	Pneumonia and bacteremia	Phlegm	Trimethoprim/sulfamethoxazole
Van Duin (2011) [12]	Male	60	Multiple sclerosis, PEG tube placement, neurogenic bladder requiring urinary diversion and ileal conduit, recurrent UTIs, nephrolithiasis, and anaphylactoid reaction to penicillins		Mechanical ventilation			Bronchoalveolar lavage	Fosfomycin, trimethoprim/sulfamethoxazole, successful prophylaxis regimen for recurrent UTIs
Umar et al. (2022) [3]	Male	89	Poorly draining Foley and hematuria along with suprapubic pain	Prostate adenocarcinoma on androgen deprivation therapy, bladder wall mass status post biopsy showing invasive high-grade urothelial carcinoma with extensive necrosis, urinary retention status post transurethral resection of the prostate and placement of an indwelling urinary catheter, right-sided hydronephrosis status post right percutaneous nephrostomy tube placement, hypothyroidism, hypertension, and prediabetes	Foley catheter			Collected from indwelling urinary catheter	Trimethoprim/sulfamethoxazole, ceftazidime

Discussion

Stenotrophomonas maltophilia is an emerging nosocomial infection that has now been recognized worldwide [2]. UTIs caused by the pathogen are rare, but they may cause significant morbidity. Several risk factors are associated with the severe disease caused by this pathogen, including underlying malignancies, immunocompromised state, indwelling catheter, prolonged antibiotic treatment, and prolonged ICU stay [2,3,13]. Trimethoprim-sulfamethoxazole (TMP-SMX) has generally been the mainstay of treatment; however, some cases of resistance have been reported. A case reported by Savini et al. [9] was sensitive to only chloramphenicol and rifampin. The development of resistance in this organism may be partly explained by the formation of biofilms on indwelling catheters [9,14], partially due to the formation of β-lactamases, such as penicillinase (L1), cephalosporin (L2) [14], aminoglycoside acetyltransferase [14], and SmeDEF pump formation [9,14]. In view of these multiple modes of antibiotic resistance formation, providing appropriate treatment can be a significant challenge.

The severe disease form of this condition is associated with significant morbidity and mortality. Risk factors for severe disease include severe septic shock, malignancy, and end-organ failure [2]. In our review, we noted that patients who developed the infection and subsequent severe illness as a result of UTI had some underlying urological or nephrological disease process, which, in addition to other risk factors, contributed to the development of a UTI. In the case reported by Vaidyanathan et al., there was underlying renal large renal calculus, which, in combination with the placement of nephrostomy tubes and double J stents, may have led to pyonephrosis for which pigtail catheter placement was required [8]. The case report by Petca et al. describes the development of a severe left-sided pyonephrosis requiring exploratory lumbotomy with left-sided nephrectomy and the evacuation of 200 cc of pus. An underlying J stent was thought to be associated with a high-grade hydroureteronephrosis. Additional risk factors reported in these cases include underlying urothelial carcinoma [3,11], neurogenic bladder [12], and permanent bladder catheterization in patients with myelofibrosis [9]. These patients were treated based on their sensitivities, the majority of whom were susceptible to TMP-SMX. Patients in all of these cases suffered significant morbidity, including prolonged hospital and intensive care unit stays and invasive surgical procedures.

Direct contact with the source of the disease is usually the mode of transmission for this disease [2]. Several cases of disease transmission have been reported through direct contact with healthcare professionals [2,15]. The pathogens were also found in hospital tap water, which is an important source of infection [16]. The practice of adequate hand hygiene and the avoidance of hospital tap water should be maintained among patients with comorbidities and relevant risk factors to prevent the development of this disease. The prevention of biofilms on indwelling catheters should be a primary focus in order to prevent the development of this disease.

The authors do acknowledge the limitations of the study, particularly the small sample size of the reported cases. We recommend larger retrospective studies to gain more insights into the topic.

## Conclusions

Stenotrophomonas maltophilia is increasingly being reported as a cause of UTIs. It is crucial to understand the risk factors associated with the disease as patients with certain comorbidities can develop a more serious illness. Emphasis should be placed on understanding the mechanism of antibiotic resistance so as to devise an appropriate antibiotic regimen to treat the infection. Educating the hospital staff on the possible modes of prevention is vital to preventing the spread of the disease.
